# Hydrogen peroxide induced genomic instability in nucleotide excision repair-deficient lymphoblastoid cells

**DOI:** 10.1186/2041-9414-1-16

**Published:** 2010-12-22

**Authors:** Kalpana Gopalakrishnan, Grace Kah Mun Low, Aloysius Poh Leong Ting, Prarthana Srikanth, Predrag Slijepcevic, M Prakash Hande

**Affiliations:** 1 Department of Physiology, Yong Loo Lin School of Medicine, National University of Singapore, 117597, Singapore; 2 Division of Biosciences, School of Health Sciences and Social Care, Brunel University, Uxbridge UB8 3PH, UK

## Abstract

**Background:**

The Nucleotide Excision Repair (NER) pathway specialises in UV-induced DNA damage repair. Inherited defects in the NER can predispose individuals to Xeroderma Pigmentosum (XP). UV-induced DNA damage cannot account for the manifestation of XP in organ systems not directly exposed to sunlight. While the NER has recently been implicated in the repair of oxidative DNA lesions, it is not well characterised. Therefore we sought to investigate the role of NER factors Xeroderma Pigmentosum A (XPA), XPB and XPD in oxidative DNA damage-repair by subjecting lymphoblastoid cells from patients suffering from XP-A, XP-D and XP-B with Cockayne Syndrome to hydrogen peroxide (H_2_O_2_).

**Results:**

Loss of functional XPB or XPD but not XPA led to enhanced sensitivity towards H_2_O_2_-induced cell death. XP-deficient lymphoblastoid cells exhibited increased susceptibility to H_2_O_2_-induced DNA damage with XPD showing the highest susceptibility and lowest repair capacity. Furthermore, XPB- and XPD-deficient lymphoblastoid cells displayed enhanced DNA damage at the telomeres. XPA- and XPB-deficient lymphoblastoid cells also showed differential regulation of XPD following H_2_O_2 _treatment.

**Conclusions:**

Taken together, our data implicate a role for the NER in H_2_O_2_-induced oxidative stress management and further corroborates that oxidative stress is a significant contributing factor in XP symptoms. Resistance of XPA-deficient lymphoblastoid cells to H_2_O_2_-induced cell death while harbouring DNA damage poses a potential cancer risk factor for XPA patients. Our data implicate XPB and XPD in the protection against oxidative stress-induced DNA damage and telomere shortening, and thus premature senescence.

## Background

The nucleotide excision repair (NER) pathway is a versatile DNA repair mechanism that recognizes and efficiently removes an array of structurally diverse DNA lesions including ultraviolet (UV)-induced lesions, intra-strand crosslinks and bulky chemical adducts such as those induced by compounds in tobacco smoke. The NER comprises of more than three dozen genes working in spatial and temporal concert and is differentiated into two sub-pathways - the global genome-NER (GG-NER) and transcription coupled repair (TCR) - that differ only in damage recognition [1,2].

Inherited defects in the NER predispose an individual to genetic disorders featuring genomic instability and segmental progeria - Xeroderma pigmentosum (XP), Cockayne syndrome (CS) and Trichothiodystrophy (TTD). XP is a rare autosomal recessive congenital disorder that arises from mutations in XP proteins, XPA - XPG, and a variant form XPV. XP patients are predisposed to sun-induced cutaneous cancer incidence by more than a thousand-fold, display sunlight hypersensitivity, high frequency of internal tumours, accelerated neurodegeneration and developmental abnormalities [3,4]. XPA, XPB and XPD are three proteins that play pivotal roles in both the GG-NER and TCR. XPA is involved in DNA damage recognition through site-directed binding of rigidly kinked double stranded DNA, thereby engaging the excision of the lesion [5,6]. XPB and XPD unwind the local area of the damaged DNA; by virtue of constituting transcription factor II H (TFIIH), which is part of the RNA Polymerase II holocomplex, they are important not only for repair but also for basal transcription although the helicase activity of XPD is dispensable for transcription [7]. Mutations compromising the function of either of these XP genes can lead to specific clinical outcomes. In particular, mutations in XPA results in only XP while mutations in either XPB or XPD can result in XP, XP/CS, TTD or XP/TTD. Additionally, polymorphisms in XP genes can give rise to diseases with phenotypic heterogeneity of differing severities [8-10].

Although a common denominator for lesions repaired by the NER is the presence of significant distortion of the DNA helix [11], it has more recently been implicated in the repair of minor oxidative base damages that are not helix distorting [12]. Despite the base excision repair (BER) being the main pathway for the repair of such lesions, the NER is also important and may serve as a back-up system [13,14]. Endogenous oxidative damage occurs via the by-production of reactive oxygen species (ROS) such as hydrogen peroxide (H_2_O_2_) during normal cellular metabolism. Oxidative DNA damage constitutes strand breaks, helical distortions and hindrance to base pairing, all of which alter important genetic information by interfering with replication and transcription. Accumulation of oxidative lesions thus compromises DNA integrity predisposing to cancer [15,16] and ageing [17].

UV-induced damage cannot account for all the symptoms of XP and related disorders, especially those in organ systems not directly exposed to sunlight. A class of oxidative lesions has been shown to be specifically repaired by the NER and may play a role in neurodegeneration in XP patients [18,19]. In fact, oxidative base lesions have been implicated in patients suffering from XP/CS who display the skin diseases of XP together with the somatic and neurological abnormalities of CS [20]. Further, the neurological symptoms of XP/CS may have resulted from aberrant DNA repair of lesions induced endogenously during oxidative metabolism [8].

Oxidative stress through ROS generation is downstream to various other genotoxic agents including UV-irradiation [17]. Hence oxidative stress appears to be an important player in the development of XP symptoms that do not result directly from UV exposure. In this context a recent study has suggested that TTD and XP/CS mammalian fibroblasts that lack XPD were defective in oxidative DNA lesion-repair [21]. We have previously shown that lack of functional XPA in primary human fibroblasts increased the susceptibility of sodium arsenite- and H_2_O_2_-induced genotoxicity while retaining cell viability, posing as a risk factor for cancer in XPA patients [22]. In addition, we have found that XPB dysfunction also inclined cells to genome instability and telomere shortening when exposed to H_2_O_2 _[23]. This study investigates the role of XPA, XPB and XPD in maintaining the genomic stability of lymphoblastoid cells under H_2_O_2_-induced oxidative stress.

## Materials and methods

### Cell Culture and Treatment Conditions

Epstein-Barr transformed human diploid B-lymphocytes (lymphoblastoid cells) from a normal individual (Normal-L GM03798) and patients with Xeroderma Pigmentosum (XP) Complementation Group A (XPA-L GM02344), XP Complementation Group B also exhibiting Type 2 Cockayne's Syndrome (XPB-L GM02252) and XP complementation Group D (XPD-L GM11612) were purchased from *Coriell Cell Repository, U.S.A*., and cultured in Roswell Park Memorial Institute (RPMI) 1640 medium (*Gibco Invitrogen Corporation, U.S.A.*) supplemented with 15% foetal bovine serum (FBS; *Hyclone, U.S.A.*) and 1% L-Glutamine (*Gibco Invitrogen Corporation, U.S.A.*). Cells were grown and maintained in log phase in a humidified 5% CO_2 _incubator at 37°C. Unless otherwise stated, exponentially growing cells were seeded at a density of 4 × 10^5 ^per mL in 6-well plates (*Nunc, Denmark*) and exposed to between 20 μM and 100 μM of H_2_O_2 _(*Kanto Chemical Co. Inc., Japan*) for 2 hours, following which the medium was replaced with fresh medium for a 22-hour recovery period. Such concentrations of H_2_O_2 _have been shown to be of low to no cytotoxicity with some genotoxicity [22,24].

### Cell Viability by 3-[4,5-Dimethylthiazol-2-yl]2,5-diphenyl-tetrazolium bromide (MTT) Assay

The tetrazolium salt, MTT, is reduced to a water-insoluble coloured formazan only by metabolically active cells. The relative number of viable cells can be measured following the solubilisation and quantification of the formazan salt. Suspension cells were collected and centrifuged at 260 g and resuspended in equal volume of fresh media. Fifty microlitres of the cell suspension was added in triplicates to a 96-well plate (*Nunc*) to an equal volume of 4 mg/mL MTT (*Sigma*) solution. The plate was incubated at 37°C in the dark for 2 hours before being centrifuged at 1620 g. The supernatant was carefully aspirated and the formazan formed was dissolved in 200 μL 5% v/v Sorenson's glycine buffer (0.1 M glycine (*Univar, New Zealand*), 0.1 M NaCl, pH 10.5) in dimethyl sulfoxide (DMSO; *MP Biomedicals Inc., France*), following which the dissolved salt was measured at 570 nm using μQuant Microplate Spectrophotometer. Data is represented as the percentage of absorbance of the sample over the untreated counterpart.

### Analysis of Cell Cycle by Fluorescence Activated Cell Sorting (FACS)

Harvested cells were washed in 1 × PBS, fixed in 70% ethanol: 1 × PBS and stained with 2 mg propidium iodide (PI): 2 mg RNase A (*Roche, U.S.A.*)/100 mL 0.1% BSA in 1 × PBS for 30 minutes at 37°C in the dark. Samples were analysed by flow cytometry at 488 nm excitation λ and 610 nm emission λ. Approximately 20,000 events were collected and the data obtained was analysed using WINMDI software.

### Cytokinesis Blocked Micronucleus (CBMN) Analysis

Immediately after the 2-hour H_2_O_2 _treatment, cells were incubated in fresh medium with 4.0 μg/mL cytochalasin B (*Sigma*) for 22 hours. The protocol used is adapted from [25] and cells were processed as described before [22,26]. One thousand binucleated cells with/without the presence of micronuclei were scored under an Axioplan 2 imaging fluorescence microscope (*Carl Zeiss, Germany*) with an appropriate triple band filter.

### Chromosome Aberration (CA) Analysis

Following 2-hour H_2_O_2 _treatment, cells were allowed to grow in fresh medium for 24 hours, then arrested at metaphase with 10 μL/mL karyomax colcemid solution (*Gibco*) for 4 hours before being subjected to hypotonic swelling in pre-warmed 0.075 M KCl at 37°C for 12 minutes and fixation with Carnoy's fixative (3 parts methanol: 1 part glacial acetic acid). Fluorescent *in situ *hybridisation and analysis were performed as described before [22,26] using Cy3-labelled PNA-telomere and FITC-labelled PNA-centromere probes (*Applied Biosystems, U.S.A.*) [27].

### Alkaline Single Cell Gel Electrophoresis (Comet) Assay

Two sets of cells were plated for the comet assay. Following treatment, one set was allowed to undergo a recovery period of 22 hours in fresh media without H_2_O_2 _whilst the other was harvested immediately. Cells were processed as described before [22]. One hundred randomly selected cells were examined per sample using Comet Imager Software (*Metasystems, Germany*). Extent of DNA damage was expressed as a measure of comet tail moments, which corresponds to the fraction of DNA in the comet tail.

### Western Blotting

Cells were harvested at the same time points as that of the Comet assay (see above), lysed in 10 mM Tris-HCl (pH 7.4)/1% SDS/1 mM sodium ortho-vanadate (*Sigma*)), homogenised with a 291/2" syringe to shear DNA and centrifuged at 16,100 g at 4°C for 6 minutes. The supernatant was collected and estimated for protein concentrations using microBCA™ Protein Assay Kit (*Pierce*) according to manufacturer's instructions. An equal amount of protein (~40 μg) was resolved in SDS-polyacrylamide gels before electro-blotting onto nitrocellulose membranes (*Biorad Co.*). Membranes were blocked at room temperature for 1 hour with 5% (v/w) non-fat milk powder in TBS-T (0.1% v/v Tween-20 (*Sigma*) in 1×TBS (10 mM NaCl, 10 mM Tris pH 7.4 in dH_2_O)), incubated with primary antibodies specific for the protein of interest, washed in TBS-T to remove excess antibody, incubated in secondary antibodies specific for the primary antibody at room temperature for 1 hour, then washed in TBS-T to remove excess antibody. Primary antibodies used were the mouse monoclonal antibodies for p53 (DO-1, 1:1,000), phospho-p53 (Ser 15 16G8, 1:1000; *Cell Signaling Technology, Inc., U.S.A.*), p21 (F-5, 1:500), survivin (D-8, 1:200), PCNA (PC-10, 1:500) and XPD (184.7, 1:100), rabbit polyclonal antibodies for XPA (FL-273, 1:100) and XPB (S-19, 1:100), and goat polyclonal antibody for actin (I-19, 1:2000). Secondary antibodies used were goat anti-mouse Ig G (H+L)-HRP (1:5,000), sheep anti-rabbit Ig G (H+L)-HRP (1:5,000) or donkey anti-goat Ig G (H+L)-HRP (1:5,000) depending on the primary antibody used. All antibodies unless otherwise stated were purchased from *Santa Cruz, U.S.A.*. Protein bands were visualized against a protein marker (*Biorad*) after the membranes were incubated with Supersignal^® ^West Pico Chemiluminescent Substrate (Thermo scientific, *U.S.A*) and exposed to X-ray films (*Pierce*).

### Statistical Analysis

Statistical significance between and among data sets was assessed using two-way ANOVA with Graphpad Prism. The difference was considered to be statistically significant when *^/#^p < 0.05; **^/##^p < 0.01; and ***^/###^p < 0.001.

## Results

### Hydrogen peroxide exposure resulted in loss of viability in XPB-L and XPD-L but not XPA-L cells

**Figure 1:**
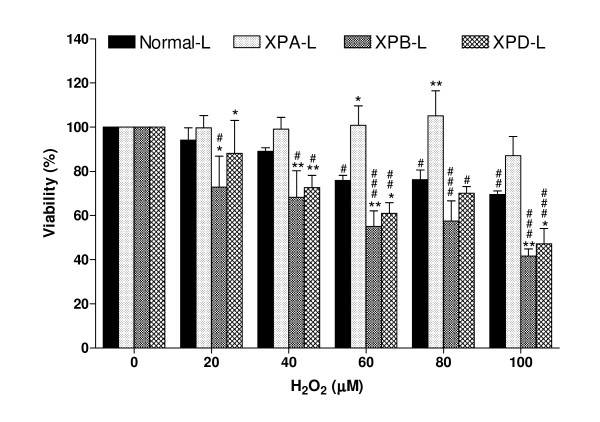
**Cell Viability**. Dose-dependent decrease in viability in Normal-L, XPB-L and XPD-L lymphoblastoid cells but not in XPA-L lymphoblastoid cells following H_2_O_2 _treatment. ^#^p < 0.05; ^##^p < 0.01; ^###^p < 0.001 compared to untreated counterparts. At higher concentrations, XPA-L exhibited significantly higher viability than Normal-L. On the contrary, XPB-L and XPD-L both exhibited significantly lower viability than Normal-L following treatment. *p < 0.05; **p < 0.01 compared to Normal-L. It is interesting to note that both XPB-L and XPD-L were also significantly less viable than XPA-L at 60 μM and higher of H_2_O_2_.

While the control lymphoblastoid cells, XPB-L, and XPD-L cells exhibited a dose-dependent decrease in viability (p < 0.05; Figure 1), XPA-L cells did not exhibit a dose-dependent change in viability following treatment. Furthermore, XPA-L cells exhibited significantly higher viability (p < 0.05) than Normal-L cells at H_2_O_2 _concentrations of 60 μM and above. XPB-L and XPD-L cells on the other hand, were significantly more sensitive to H_2_O_2 _treatment than Normal-L cells even at the low dose of 20 μM (p < 0.05). Both XPB-L and XPD-L also exhibited a significant decrease in viability than untreated counterparts following treatment at doses of 40 μM H_2_O_2 _and above (p < 0.05), while Normal-L cells exhibited significant sensitivity to H_2_O_2 _compared to untreated counterparts only from doses as high as 80 μM (p < 0.05). It is also interesting to note that XPB-L and XPD-L cells were more sensitive to H_2_O_2_-induced oxidative stress than XPA-L cells. These differences in viability response between XP-deficient cell types prompted our investigation of cell cycle profiles by PI staining.

### XPB-L and XPD-L cells exhibited an increase in the sub-G1 population, while XPA-L cells demonstrated a dose-dependent increase in the G2/M population

The primary purpose of cell cycle analysis was to observe changes in the sub-G1 population of cells, which would indicate the occurrence of early necrosis and/or apoptosis.

**Figure 2:**
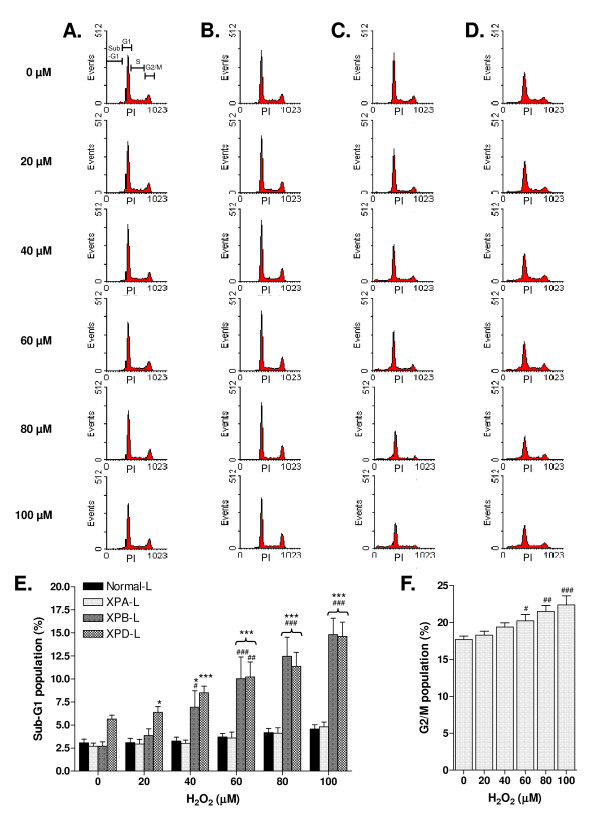
**Cell cycle profiles of A. Normal-L, B. XPA-L, C. XPB-L, and D. XPD-L lymphoblastoid cells following H**_**2**_**O**_**2 **_**treatment**. PI: Propidium iodide, indicating amount of DNA stained per cell. The top profile of panel A depicts an illustration of the distribution of cells in each phase of the cell cycle (sub G1: apoptotic cells; G1: G1-phase cells; S: S-phase cells; G2/M: G2/M-phase cells). **E**. Percentage of sub-G1 population. **F**. Percentage of G2/M population in XPA-L cells. ^#^p < 0.05; ^##^p < 0.01; ^###^p < 0.001 compared to untreated counterparts. *p < 0.05; ***p < 0.001 compared to Normal-L. Data is representative of at least three independent experiments.

In response to treatment, cell cycle profiles of Normal-L cells did not change noticeably except for a slight increase in sub-G1 population at the higher concentrations (Figure 2A). XPB-L and XPD-L cells exhibited increases in the sub-G1 population with increasing concentrations of H_2_O_2 _(Figure 2C and 2D respectively, and Figure 2E). It is further noteworthy that XPB-L cells displayed a corresponding decrease in percentage of cells in the G1 and G2/M phases following treatment. XPA-L cells on the other hand, did not demonstrate changes in the sub-G1 population but exhibited a dose-dependent increase in the G2/M population (Figure 2B, E and 2F).

### XP-deficient lymphoblastoid cells exhibited significantly higher DNA damage than Normal-L cells following H_2_O_2 _exposure

We were next interested to investigate whether the lymphoblastoid cells sustained DNA damage from H_2_O_2 _exposure in the absence of cell cycle arrest. The extent of DNA damage accumulated in XP-deficient lymphoblastoid cells in response to H_2_O_2_-induced oxidative stress was studied using three DNA markers - CBMN, CA and comet.

**Figure 3:**
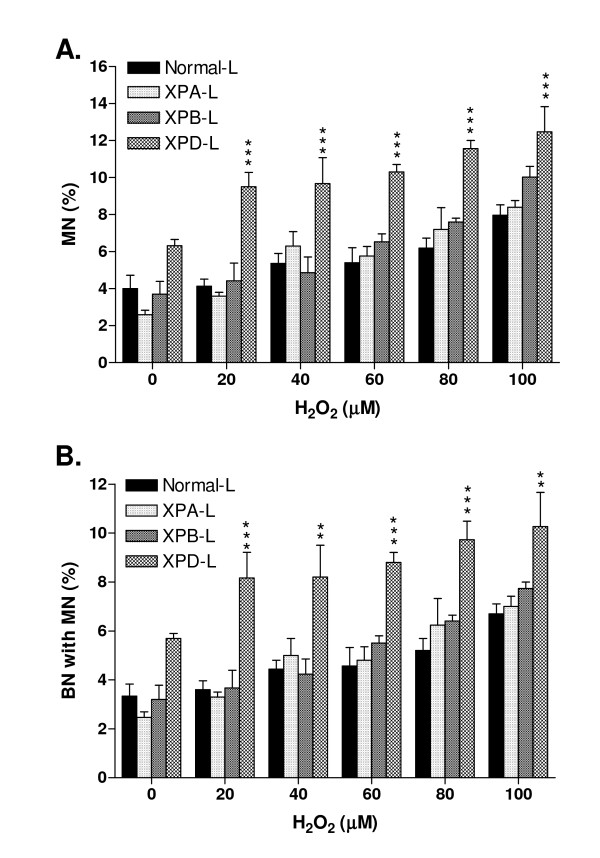
**Cytokinesis Blocked Micronucleus (CBMN) Analysis: A. Percent MN per 1000 BN scored**. **B**. Percent BN with presence of MN. There were dose-dependent increases in percent MN per 1000 BN as well as in percent BN with presence of MN in all lymphoblastoid cells. XPA-L and XPB-L both did not show significant increase in damage markers compared to Normal-L. XPD-L, however, exhibited significantly higher damage markers not only than Normal-L lymphoblastoid cells, but XPA-L and XPB-L as well. **p < 0.01, ***p < 0.001 compared to Normal-L. Two-way ANOVA.

Binucleated (BN) cells were scored for the presence and distribution of micronuclei (MN). The frequency of MN increased significantly in a dose-dependent manner in all cell types (p < 0.05; Table 1). XPA-L and XPB-L cells did not exhibit significant increase in percent MN and percent BN with MN compared to Normal-L cells (p > 0.05) while XPD-L cells showed significant increase in these damage markers from H_2_O_2 _doses as low as 20 μM (p < 0.001; Figure 3). Clearly XPD-L cells were the most sensitive as they also showed significantly more percent MN compared to their untreated counterparts even at 20 μM H_2_O_2 _(p < 0.05; Table 1) whereas XPA-L and XPB-L cells exhibited such increase only at 40 μM and 60 μM H_2_O_2 _respectively (p < 0.05), and Normal-L cells displayed this increase only at 100 μM H_2_O_2 _(p < 0.01). It is noteworthy that XPD-L cells also exhibited the highest basal MN frequency as opposed to all other cells (Table 1; Figure 3).

**Table 1:** Cytokinesis-blocked micronucleus assay

		Number of MN						
				
Cells	**H**_**2**_**O**_**2 **_**(μM)**	1	2	3	4	>4	% MN	% BN with MN
	0	2.80 ± 0.72	0.33 ± 0.12	0.13 ± 0.06	0.03 ± 0.06	0	4.00 ± 1.25	3.33 ± 0.86
	
	20	3.07 ± 0.60	0.47 ± 0.12	0	0.03 ± 0.06	0	4.13 ± 0.67	3.60 ± 0.62
	
Normal-L	40	3.63 ± 0.46	0.60 ± 0.27	0.13 ± 0.12	0.03 ± 0.06	0	5.37 ± 0.92	4.43 ± 0.64
	
	60	3.83 ± 1.21	0.53 ± 0.31	0.17 ± 0.06	0	0	5.40 ± 1.41	4.57 ± 1.30
	
	80	4.37 ± 0.74	0.57 ± 0.15	0.23 ± 0.06	0	0	6.20 ± 0.92	5.20 ± 0.85
	
	100	5.67 ± 0.46	0.87 ± 0.21	0.13 ± 0.06	0	0.03 ± 0.06	7.97 ± 0.96**	6.70 ± 0.70**

	0	2.33 ± 0.42	0.13 ± 0.15	0	0	0	2.60 ± 0.40	2.47 ± 0.38
	
	20	3.00 ± 0.35	0.30 ± 0.00	0	0	0	3.60 ± 0.35	3.30 ± 0.35
	
XPA-L	40	4.03 ± 1.20	0.80 ± 0.10	0.10 ± 0.10	0	0.07 ± 0.06	6.30 ± 1.35**	5.00 ± 1.20*
	
	60	4.00 ± 1.14	0.67 ± 0.25	0.10 ± 0.00	0.03 ± 0.06	0	5.77 ± 0.90*	4.80 ± 0.95
	
	80	5.43 ± 1.92	0.63 ± 0.21	0.17 ± 0.21	0	0	7.20 ± 2.04***	6.23 ± 1.89***
	
	100	5.90 ± 0.95	0.83 ± 0.12	0.23 ± 0.21	0.03 ± 0.06	0	8.40 ± 0.62***	7.00 ± 0.72***

	0	2.77 ± 0.95	0.43 ± 0.15	0	0	0	3.70 ± 1.20	3.20 ± 1.01
	
	20	3.10 ± 1.15	0.47 ± 0.21	0.10 ± 0.10	0	0	4.43 ± 1.65	3.67 ± 1.26
	
XPB-L	40	3.77 ± 0.85	0.37 ± 0.15	0.10 ± 0.10	0	0	4.87 ± 1.47	4.23 ± 1.08
	
	60	4.83 ± 0.55	0.43 ± 0.15	0.23 ± 0.12	0	0	6.53 ± 0.74*	5.50 ± 0.52
	
	80	5.57 ± 0.42	0.60 ± 0.10	0.23 ± 0.12	0	0	7.60 ± 0.35**	6.40 ± 0.44**
	
	100	6.00 ± 0.36	1.37 ± 0.15	0.37 ± 0.23	0	0	10.03 ± 1.00***	7.73 ± 0.46***

	0	5.10 ± 0.17	0.57 ± 0.12	0.03 ± 0.06	0	0	6.33 ± 0.58	5.70 ± 0.35
	
	20	7.07 ± 2.11	0.90 ± 0.17	0.17 ± 0.15	0.03 ± 0.06	0	9.50 ± 1.35*	8.17 ± 1.82
	
XPD-L	40	7.03 ± 2.14	0.90 ± 0.10	0.23 ± 0.21	0.03 ± 0.06	0	9.67 ± 2.44*	8.20 ± 2.25
	
	60	7.60 ± 0.62	0.90 ± 0.20	0.30 ± 0.17	0	0	10.30 ± 0.70**	8.80 ± 0.70**
	
	80	8.23 ± 1.56	1.20 ± 0.36	0.27 ± 0.21	0.03 ± 0.06	0	11.57 ± 0.76	9.73 ± 1.29***
	
	100	8.70 ± 2.19	1.17 ± 0.38	0.30 ± 0.10	0.07 ± 0.06	0.03 ± 0.00	12.47 ± 2.37***	10.27 ± 2.42***

Micronuclei (MN) scores, percentage of binucleated cells (BN) with MN and percentage of MN in cytokinesis-blocked binucleated lymphoblastoids following H_2_O_2 _treatment. Data is represented as mean ± SD. Statistics shown only for last two columns where *p < 0.05, **p < 0.01, ***p < 0.001, where significance is calculated to the respective cell type's untreated counterpart.

**Figure 4:**
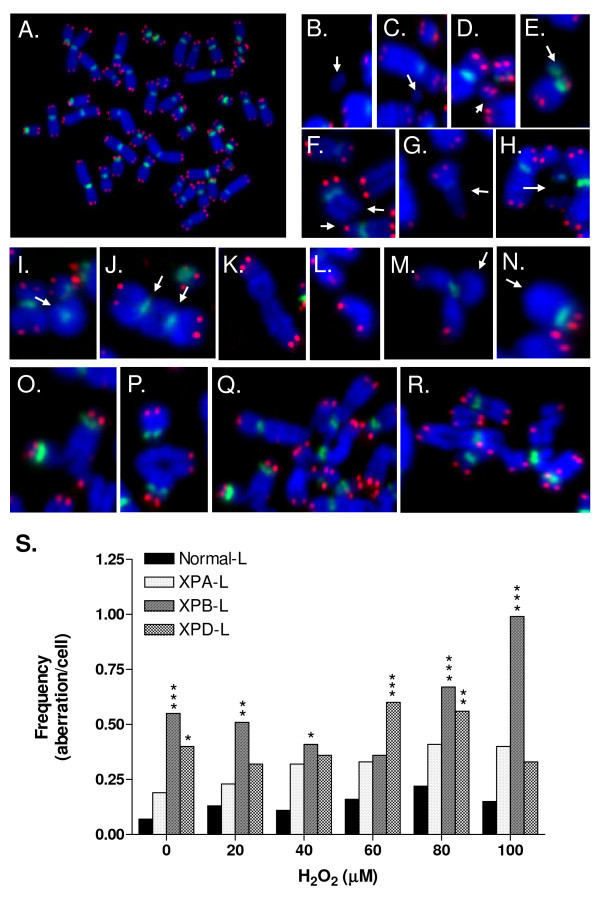
**Telomere PNA-FISH was used to detect chromosomal aberrations (CA) in metaphases**. **A-R**. Cy3-telomere and FITC-centromere PNA probes, depicted by the red and green signals respectively, and DAPI (blue) counterstain were used. Cell type and H_2_O_2 _concentrations used on the cells are stated within the parentheses. **A**. Metaphase spread with 46 chromosomes and no detected aberrations (XPA-L, 20 μM). **B-H**. Aberrations categorized as breaks. Interstitial deletion resulting in a **B**. double minute (XPD-L, 0 μM) and **C**. acentric fragment (XPD-L, 0 μM). **D**. Terminal break in chromosome resulting in an acentric fragment with telomere signals (XPA-L, 60 μM). **E**. Interstitial deletion resulting in a centric fragment (XPA-L, 80 μM). **F**. Undetected telomere signals (XPB-L, 0 μM). **G**. Chromatid break (XPD-L, 80 μM). **H**. Chromosome break (Normal-L, 80 μM). **I-N**. Aberrations categorized as simple fusions. **I**. Centric ring (XPA-L, 100 μM). **J**. Dicentric chromosome with both centromeres depicted by white arrows (XPB-L, 100 μM). **K**. Fusion of two terminally broken chromosomal fragments forming a large chromosome-like structure (XPD-L, 40 μM). **L**. Fusion of two terminally broken chromatid fragments (Normal-L, 20 μM). **M-N**. Sister chromatid fusions (Normal-L, 20 μM and XPD-L, 60 μM respectively). **O-R**. Aberrations categorized as complex fusions. **O-P**. Triradial-like structures (XPA-L, 60 μM and XPD-L, 60 μM respectively). **Q-R**. Quadiradial-like structures (both from XPA-L, 60 μM). **S**. Frequency of total aberration per spread following H_2_O_2 _treatment. *p < 0.05; **p < 0.01, ***p < 0.001 compared to Normal-L.

Chromosomal aberrations (CA) such as fragments, breaks and fusions sustained by the lymphoblastoid cells following H_2_O_2 _exposure are shown in Figure 4. The scores and distribution of CA are represented in Table 2 and Figure 4S. H_2_O_2 _treatment led to an overall increase in CA in all cell types. Notably, all three XP-deficient cell types exhibited a much larger increase in total CA -specifically more breaks - than Normal-L cells following treatment (Table 2). This increase was significant in XPB-L (p < 0.05) and XPD-L (p < 0.01) but not in XPA-L cells (p > 0.05; Figure 4S); XPA-L cells appeared to be the least sensitive among the three XP-deficient lymphoblastoid cells although they exhibited more fragments than XPB-L cells (Table 2). XPB-L cells seemed to be the most sensitive to H_2_O_2_-induced CA particularly undetected telomeres (UTs) and fusions (Table 2). XPD-L cells also showed more UTs than XPA-L and Normal-L cells (Table 2).

**Table 2:** Analysis of metaphase spreads of lymphoblastoids following H2O2 treatment

			Fusions						Breaks					
					
Cell Type	**H**_**2**_**O**_**2 **_**(μM)**	Chromosomes per metaphase	T/QLS	SCF	D/T	R	EAF	Total Fusions	CB	F	UT	Total Breaks	Total Aberrations	Aberrant cells (%)
	0	45.90 ± 0.68 (44-48)	0	0	0	0	0	0	0	1	6	7	7	7
	20	46.04 ± 0.49 (44-48)	0	1	0	0	1	2	0	1	10	11	13	10
Normal-L	40	45.82 ± 0.73 (44-48)	0	0	0	0	0	0	0	3	8	11	11	9
	60	45.89 ± 0.77 (44-48)	0	0	0	0	0	0	0	5	11	16	16	14
	80	45.79 ± 0.86 (44-48)	0	0	0	1	0	1	2	11	8	21	22	14
	100	46.03 ±0.76 (44-48)	0	0	1	0	0	1	0	3	11	14	15	13

	0	45.83 ± 0.67 (44-48)	0	0	0	0	0	0	0	10	9	19	19	14
	20	45.79 ± 0.64 (44-48)	0	0	0	1	0	1	1	15	6	22	23	14
XPA-L	40	45.69 ± 0.99 (43-48)	0	1	2	0	1	4	2	13	13	28	32	22
	60	45.65 ± 0.79 (43-48)	3	0	1	1	0	5	2	12	14	28	33	22
	80	45.35 ± 1.46 (41-48)	0	0	1	0	0	1	0	22	18	40	41	26
	100	45.77 ± 0.67 (44-48)	0	0	3	1	0	4	5	16	15	36	40	28

	0	46.49 ± 0.97 (44-48)	0	1	0	0	0	1	1	5	48	54	55	39
	20	46.28 ± 1.08 (44-48)	0	1	1	0	0	2	0	5	44	49	51	37
XPB-L	40	46.61 ± 0.89 (44-48)	0	1	0	0	0	1	0	6	34	40	41	29
	60	46.53 ± 0.87 (44-48)	0	0	0	0	0	0	1	1	34	36	36	30
	80	46.43 ± 0.96 (44-48)	0	1	4	0	1	7	1	8	51	60	67	40
	100	46.42 ± 1.01 (44-48)	0	2	4	0	5	11	0	11	77	88	99	45

	0	45.86 ± 0.87 (44-48)	0	0	0	0	0	0	3	21	16	40	40	30
	20	45.86 ± 0.87 (44-48)	0	0	0	0	0	0	0	13	19	32	32	23
XPD-L	40	45.75 ± 0.86 (44-48)	0	0	1	0	1	2	0	19	15	34	36	19
	60	45.95 ± 0.86 (44-48)	2	1	1	0	0	4	6	19	31	56	60	37
	80	45.90 ± 0.91 (44-48)	0	0	1	0	0	1	1	20	34	55	56	31
	100	45.80 ± 1.10 (44-48)	0	1	1	1	0	3	3	10	17	30	33	25

T/QLS-Triradial/Quadiradial-like structures; SCF-Sister Chromatid Fusions; D/T-Di/Tricentrics; R-Rings; EAF - End-to-end fusions of acentric fragments; CB-Chromsosome/Chromatid breaks; F-fragments including double minutes, terminal acentric fragments, interstitial acentric fragments and centric fragments; UT-Undetected telomeres. Data is represented as total number of aberrations. A total of 100 spreads were analyzed in two independent experiments. *p < 0.05, **p < 0.01, ***p < 0.001, where significance is calculated to the respective cell type's untreated counterpart.

### Lack of functional XP proteins lead to reduced capacity to repair oxidative DNA lesions in XPD-L, but not XPA-L and XPB-L cells

**Figure 5:**
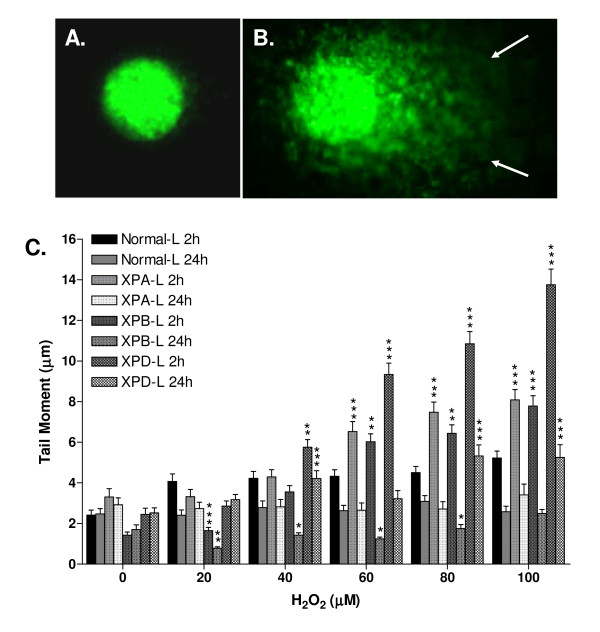
**Single Cell Gel Electrophoresis**. SYBR-Green stained **A**. comet showing apparently no damage depicted by the round head and **B**. comet with tail depicted by arrows indicating presence of damage. **C**. Tail moments immediately following 2-hour H_2_O_2 _treatment and 22-hour recovery in fresh medium. H_2_O_2 _concentrations of 60 μM and above resulted in significantly increased tail moments at 2 hours (p < 0.001) which decreased significantly following recovery (p < 0.05) in all lymphoblastoid cells. While there was no significant difference in moments between untreated Normal-L and XP-deficient lymphoblastoid cells (p > 0.05), XP-deficient lymphoblastoid cells exhibited increased tail moments when compared to Normal-L at 2 hours of H_2_O_2 _exposure. Following recovery, however, only XPD-L showed significantly higher moments than Normal-L (p < 0.001), while XPB-L showed significantly lower moments. *p < 0.05, **p < 0.01 and ***p < 0.001 indicate significantly greater tail moments when comparing XP-deficient lymphoblastoid cells to Normal-L counterparts. Data is represented as mean ± SE.

The ability of cells to recover from oxidative genotoxic assault was compared using overall genomic integrity as a parameter via the comet assay, where cells were harvested and analyzed as previously described [22]. The rationale for assessing damage at two time points was to elucidate susceptibility to oxidative DNA damage and capacity for damage-repair. Comet tail moments scored are a function of tail length and fluorescence intensity [28]. Figure 5 depicts a SYBR-Green stained nucleus showing apparently no damage (Figure 5A) and one with a comet tail indicative of DNA damage (Figure 5B).

All lymphoblastoid cells exhibited a dose-dependent increase in tail moments following the 2-hour treatment with H_2_O_2 _and this increase was significant in XP-deficient (p < 0.01) but not in Normal-L cells (p > 0.05; Figure 5C). Amongst XP-deficient cells, XPD-L cells exhibited the most drastic increase in tail moments compared to untreated and Normal-L counterparts following the 2-hour treatment, showing significant increase from 40 μM and higher doses of H_2_O_2 _(p < 0.01), while XPA-L and XPB-L lymphoblastoid cells showed significant increase in tail moment following treatment with 60 μM and higher doses of H_2_O_2 _(p < 0.01). At doses of 60 μM and above, XPD-L cells were significantly more damaged than the other two XP-deficient cell types (p < 0.05).

Post 22-hour recovery, tail moments of all lymphoblastoid cells were lower than that of the 2-hour treatment (Figure 5C), indicating repair. Notably, XPB-L cells displayed significantly lower tail moments than its untreated and Normal-L counterparts (Figure 5C) at doses as low as 20 μM H_2_O_2_, indicating capacity for repair (p < 0.01). XPD-L cells on the other hand exhibited significantly higher tail moments even after recovery compared to its untreated counterparts (p < 0.001; Figure 5A), Normal-L cells (p < 0.001) and even XPA-L and XPB-L cells (p < 0.05), indicating lowest capacity to repair damaged DNA in XPD cells as opposed to other cell types.

### XPD was differentially regulated in XPA-L and XPB-L cells following H_2_O_2 _treatment

**Figure 6:**
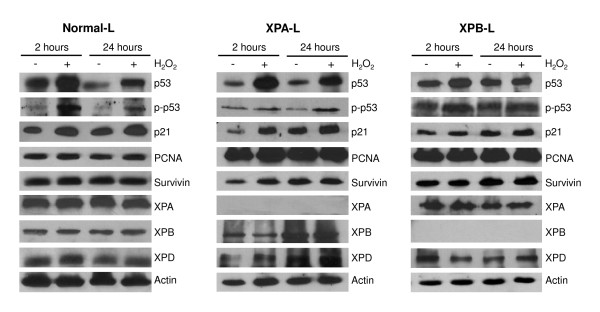
**Protein expression profiles of Normal-L, XPA-L and XPB-L cells**. p53, phospho-p53 and p21 proteins were differentially regulated in all three cell types, while PCNA, survivin, XPA, XPB and XPD proteins were not, except in XPA-L, whereby XPD exhibited differential regulation.

To gain insight into the potential role of NER in maintaining genome stability and determining cell fate upon H_2_O_2 _exposure, we investigated the expression patterns of factors involved in cell cycle, apoptosis, survival and NER in the lymphoblastoids following exposure to 60 μM H_2_O_2 _(Figure 6). This dose was chosen as Normal-L cells exhibited significantly lower viability than untreated counterparts and both XPB-L and XPD-L cells were significantly less viable than Normal-L cells at this concentration (Figure 1). Since XPB and XPD function in the same macromolecule and lymphoblastoids deficient in these proteins exhibited similar responses in our data, we explored protein expressions in only XPB-L cells.

We observed up-regulation of p53 and phospho-p53 (p-p53), and no change in PCNA and Survivin expressions in Normal-L, XPA-L and XPB-L lymphoblastoids following 2 hours of H_2_O_2 _treatment and 22 hours of recovery. p21 was up-regulated at both time points in XPA-L cells whereas it was not differentially expressed in XPB-L cells. While Normal-L cells did not show any changes in expression in the NER factors XPA, XPB and XPD, XPA-L and XPB-L cells exhibited differential regulation of XPD, where XPA-L showed up-regulation of XPD at both time points and XPB-L displayed down-regulation at 2 hours but up-regulation at recovery. As expected, XPA and XPB were not expressed in XPA-L and XPB-L cells respectively.

## Discussion

The NER has been shown to be involved in oxidative damage repair but much is still yet to be elucidated. Through the Fenton and Haber-Weiss reactions, H_2_O_2 _generates OH^- ^radicals [29,30] which inflict more than 100 different DNA modifications including 8,5'-cyclopurine-2'-deoxynucleosides (CyPudNs) [31], an oxidative lesion repaired by the NER but not the BER [18,19]. These findings suggest that unrepaired oxidative lesions may contribute to XP symptoms which cannot be accounted for by UV exposure alone such as internal cancers, developmental defects and neurodegeneration. Moreover, the NER has been suggested to be functionally linked to the BER pathway, possibly explaining compromised BER functions observed in some XP patients. Specifically, NER factors XPG and XPC have been shown to interact with and activate BER DNA glycosylases [32,33]. Here we study the possible involvement of other NER factors, XPA, XPB and XPD in oxidative lesion management.

Our cell viability and cell cycle data indicate that loss of functional XPB or XPD but not XPA leads to enhanced sensitivity towards H_2_O_2_-induced cell death in the lymphoblastoids. The dose-dependent increase in the G2/M population and lack of cell death in H_2_O_2_-exposed XPA-L cells is a phenomenon that we have previously shown in XPA-deficient primary fibroblasts [22]. XPA has been shown to interact with and to be modulated by the cell cycle checkpoint proteins ATM and ATR following irradiation [34,35]. Therefore, lack of XPA may result in aberrant signalling leading to cell cycle dysfunction and failure of apoptosis. Alternatively, since the NER pathway is not the major pathway for H_2_O_2_-induced DNA lesions, cell cycle arrest may be triggered to allow time for repair by the BER instead. The observed increased sensitivity of XPB-L and XPD-L cells to H_2_O_2_-induced cell death may possibly be attributed to the requirement of these proteins in both repair and basal transcription [36,37]. In this light, XPB and XPD are indispensible for cellular viability and only subtle mutations are found in XP, XP/CS and TTD [38,39]. However, we have previously noted that fibroblasts deficient in XPB, similar to the XPA-L lymphoblastoid cells in this study and XPA fibroblasts in our previous study, sustained genotoxic effects of H_2_O_2 _while retaining cell viability [23]. The differential results between XPB-deficient fibroblasts and lymphoblastoid cells could be due to cell type-specific responses, similar to an observation made between NER-deficient mouse embryonic stem cells and fibroblasts [40], or could have resulted from changes to the lymphoblastoids during transformation by Epstein-Barr virus.

Despite the observed differences in cell fate following H_2_O_2 _exposure, lack of functional XPA, XPB or XPD led to increased susceptibility to H_2_O_2_-induced genome instability indicative of a role of these XP factors in maintaining genomic integrity challenged by H_2_O_2_. Although lymphoblastoids deficient in XPA and XPB did not exhibit significantly more H_2_O_2_-induced MN compared to Normal-L lymphoblastoids, they demonstrated increased H_2_O_2_-induced damage susceptibility with significantly increased MN and BN per MN following treatment from lower H_2_O_2 _concentrations than that of Normal-L cells (Table 1). This is consistent with our previous findings that XPA- and XPB-deficient fibroblasts were more susceptible to H_2_O_2_-induced DNA damage [22,23].

The comet assay was used to address whether the lack of a functional XP protein compromised a cell's ability to recover from H_2_O_2_-induced DNA damage. While the comet results also reflect that deficiency in XPA, XPB or XPD increased susceptibility to H_2_O_2_-induced global DNA damage, we noted that the loss of XPD resulted in the highest susceptibility to H_2_O_2_-induced DNA damage and the lowest capacity to repair these lesions, suggesting that XPD may have a more significant role than the other two XP factors in the repair of H_2_O_2_-induced lesions. On the other hand, the increased sensitivity of these NER-deficient lymphoblastoids to H_2_O_2_-induced comet tails may be confounded by disturbed redox homeostasis and not repair-deficiency per se as seen in XPC-deficient and XPC-silenced cells [41]. Of particular interest is the tail moment recovery of XPB-L cells to lengths shorter than that of Normal-L counterparts but comparable to that of XPB-L untreated cells, suggesting that while XPB-L may be more susceptible to H_2_O_2_-induced damage, these cells do not lack the ability to repair the sustained damage. Similarly, while XPA-L cells sustained DNA damage, they exhibited competent recovery. This seems to suggest that XPA and XPB may not be integral in oxidative damage-repair. While a study supports the notion that XPB in particular is not involved in the BER [42], we have previously demonstrated the importance of XPA and XPB in the protection against oxidative DNA lesions [22,23]. Moreover, *XPB*, *XPD*, and *XPG *deficiencies have been shown to compromise general repair [12]. The difference in the responses seen in the previous and current work may be attributed to cell-type specific responses. Nonetheless, that Normal-L lymphoblastoid cells did not sustain significant damage following H_2_O_2 _exposure and exhibited competent repair mechanisms highlight the importance of XP factors in the protection against H_2_O_2_-induced oxidative DNA damage.

The marked increase in the frequency of UT in XPB-L cells suggests that XPB may have a crucial role in maintaining telomere integrity (Table 2). In line with this, we have previously shown that the lack of functional XPB leads to increased telomere attrition under oxidative stress [23]. XPD may also be integral in regulating telomere dynamics as the loss of this protein in lymphoblastoid cells also resulted in augmented frequency of UT (Table 2). The NER factor XPF was found to be implicated in telomere loss in mice overexpressing TRF2 [43], suggesting that other NER factors could play a role in telomere regulations. Moreover, XPB and XPD are part of the family of RecQ helicases which include WRN and BLM helicases shown to regulate telomere homeostasis [44-47].

Upregulation of p53 and p-p53 in Normal-L, XPA-L and XPB-L cells immediately following H_2_O_2 _exposure indicates proper DNA damage signalling responses were in place. H_2_O_2_-induced DNA damage induces the p53 pathway, which orchestrates DNA damage-repair responses, cell cycle arrest or apoptosis [48]. In response to DNA damage, p53 is upregulated and phosphorylated at Ser15 [49]. That p53 and p-p53 upregulation in XPB-L cells following 22-hour recovery is not as dramatic as that of XPA-L and Normal-L cells suggests that lack of XPB elicits only a short-lived DNA damage response, correlating with our comet data where XPB-L cells exhibited competent damage-repair. p21 is a direct downstream target of p53 and has been shown to integrate DNA damage into growth arrest or apoptotic signalling pathways [50]. XPA has shown involvement in DNA damage recognition and cell cycle checkpoints [51]. Consequently, loss of XPA may have led to aberrant signalling whereby accumulation of p53, p-p53 and p21 lead to cell cycle arrest instead of p53-dependant apoptosis. This observation correlates with our data where H_2_O_2_-exposed XPA-L cells did not lose viability but exhibited a dose dependent increase in G2/M arrest.

PCNA plays a critical role in many DNA repair processes, including the NER [52]. Although studies have revealed that the lack of functional NER does not affect PCNA ubiquitination following UV irradiation [53], whether the protein is differentially regulated in response to H_2_O_2 _treatment in an NER deficient background has not been explored. Survivin is a member of the inhibitor of apoptosis (IAP) protein family that regulates cell cycle and death, and is commonly upregulated in cancers [54]. The absence of PCNA and survivin differential expression suggests that these proteins are not involved in the processes affected by H_2_O_2 _exposure.

XPA, XPB and XPD were not observed to be differentially regulated in Normal-L cells following H_2_O_2 _exposure, indicating that they may not be the immediate effectors of this type of stress. Indeed, the major pathway for oxidative DNA damage recognition and repair is the BER, and it would be worth investigating the modulation of BER proteins immediately following H_2_O_2 _exposure. Interestingly, while XPA and XPB proteins in XPB-L and XPA-L cells respectively were not modulated following H_2_O_2 _exposure, XPD was upregulated in response to H_2_O_2 _in XPA-L cells and in XPB-L cells following recovery, suggesting that a lack of functional XPA or XPB protein may be compensated for by upregulating XPD under H_2_O_2_-induced oxidative stress. Both XPB and XPD function in the TFIIH complex and have been proposed to be involved in the DNA damage verification process, whereby both helicases scan DNA strands by moving in the same direction [55,56]. XPB-deficient cells may therefore upregulate XPD to compensate for the lack of XPB function.

## Conclusions

Our data shows that while all three XP-deficient lymphoblastoid cells unanimously exhibited increased susceptibility to H_2_O_2_-induced DNA damage, XPB and XPD, but not XPA may be involved in the repair of such lesions. While lack of functional XPA led to aberrant damage-induced cell death signalling posing a cancer risk, the lack of functional XPB and XPD led to higher damage sustenance, resulting in cell death. This could possibly explain why XPA patients develop cancer while some XPB and XPD patients are not cancer prone and manifest developmental defects of CS and TTD. Further, XPB and XPD may be involved in telomere maintenance under oxidative stress challenged by H_2_O_2_. Although to date telomere lengths of XP patients have not been fully explored, our findings link NER deficiency to telomere integrity, which may be associated with the progeroid symptoms of XP. Oxidative stress has been associated with premature senescence, accelerated ageing and telomere attrition [17,57] while telomere attrition and dysfunction have been linked to premature senescence and carcinogenesis [57]. Taken together, our data implicates a role for XP proteins in H_2_O_2_-induced oxidative stress management and further corroborates that oxidative stress is a significant contributing factor in XP symptoms, especially those that cannot be directly attributed to UV exposure.

## Competing interests

The authors declare that they have no competing interests.

## Authors' contributions

KG and GKML participated in the design of experiments, carried out the experiments, analysed the data and drafted the manuscript. APLT was involved in the experimental design, comet analysis, and manuscript preparation. PS^1 ^was involved in the cytogenetic analysis. PS^2 ^was involved in experimental design and manuscript preparation. MPH oversaw the overall experimental design and coordinated all research work and manuscript preparation. All authors read and approved the final manuscript.
